# Zinc therapy improves adverse effects of long term administration of copper on epididymal sperm quality of rats

**Published:** 2013-07

**Authors:** Homayoon Babaei, Jalil Abshenas

**Affiliations:** *Department of Clinical Sciences, Faculty of Veterinary Medicine, Shahid Bahonar University of Kerman, Kerman, Iran.*

**Keywords:** *Copper toxicosis*, *Zinc sulfate*, *Sperm*, *Rat*

## Abstract

**Background:** Industrial copper ingest is a common form of poisoning in animals. Zinc has an important role in the physiology of spermatozoa, in sperm production and viability.

**Objective:** This study was set to investigate whether the adverse effects of long term copper consumption on quality of rat spermatozoa could be prevented by zinc therapy.

**Materials and Methods:** Forty eight mature (6-8 weeks old) male rats were randomly allocated to either control (Cont, n=12) or three treatment groups each containing twelve animals. Animals in the first treatment group was gavaged with copper sulfate, the second treatment group was injected with zinc sulfate, and the third treatment group was given combined treatment of copper and zinc. Control animals received normal saline using the same volume and similar methods. Six rats from each group were sacrificed on day 28 and 56 after treatments for sperm quality evaluations.

**Results:** In spite of testicular weight reduction 56 days after copper consumption in comparison to the control group (p=0.002), there was not a significant difference between the control and combined treatment of copper and zinc group (31.40±0.55 vs. 28.63±0.55, p=0.151). Administration of copper caused a significant decrease in the sperm count, viability and motility after 56 days compared to the control group. However, a complete recovery in sperm count was seen in combined treatment of copper and zinc group after 56 days compared to the control group (p=0.999) and a partial improvement was seen about the percentage of viability and motility (p<0.001).

**Conclusion:** Adverse effects of long term consumption of copper on sperm quality could be prevented by zinc therapy in rats.

## Introduction

Long-term intake of copper (Cu) compounds of different origin is the most common form of copper poisoning in animals. It means that the animals are reared close to industrial plants, and ingest Cu from industrial deposits through feed or from air throughout their entire life (1). Another important form of Cu poisoning is occupational exposure which may lead to copper toxicosis in the industrial workers (2). Clinical manifestations associated with Cu poisoning and its pathological features specially in organs such as liver, kidney, spleen, lung and intestine have been well demonstrated in animals (1, 3). Recently, the adverse effect of Cu poisoning on sperm quality and testicular histopathology has been reported (4). 

Attempt has been made to prevent the occurrence of the disease by dietary supplementation with molybdenum and sulfate but despite of the success of these reports, there has been fear of inducing a Cu deficiency state (5, 6). Zinc (Zn) is a micronutrient that has an important role in the physiology of spermatozoa, in sperm production and/or viability, in the prevention of sperm degradation and in sperm membrane stabilization (7, 8). Increase in liver Zn content causes a redistribution of hepatic Cu, with an increase in the amount bound to metallothionein, which is thought to be involved in the storage and detoxification of copper (9). 

It has been found that zinc supplementation is able to control the incidence of Cu toxicosis in sheep (10). Therefore, in this paper, protective effect of zinc sulfate against deleterious effects of long term administration of copper on rat epididymal sperm quality has been investigated.

## Materials and methods

**Animals**

Male Sprague-Dawley rats (250-300 gr) were obtained from Razi Research Institute of Kerman, Iran. The mice were fed with standard commercial laboratory chow [(pellet form), Javeneh Khorasan Co., Mashhad, Iran] and water ad libitum and housed under standard laboratory conditions (12 h light: 12 h dark and 22±2^o^C) during the experimental period in October-December 2011. All animals were care for and used in accordance with the International Guiding Principle for Biomedical Research Involving Animals at Veterinary Faculty of Shahid Bahonar University of Kerman, Iran (Certificate No. 550/237).

**Study design**

Forty eight mature (6-8 weeks old) male rats were randomly allocated to either control (Cont, n=12) or three treatment groups each containing twelve animals. The first treatment group received copper sulfate at a dose of 200 mg/kg in 0.2 cc once a day for 56 consecutive days by gavage (Cu group, n=12), the second treatment group was injected zinc sulfate at a dose of 10 mg/kg in 0.1 cc intraperitoneally every other day for 56 days (Zn group, n=12) and the third treatment group was given combined treatment of the first and second treatment groups (ZC group, n=12). Control animals received normal saline using the same volume and similar methods. The dose of copper sulfate and zinc sulfate used in our experiment was according to the previous studies (4, 11). 

**Sperm quality**

Six male rats from the experimental groups were sacrificed upon diethyl ether anesthesia (May & Baker Ltd., Dagenham, England) by cervical dislocation on days 28 and 56 after treatments. The testes and epididymides were gently excised and weighed and the cauda epididymides were isolated and placed in a Falcon tube containing 1 mL of D-PBS (pH=7.4, mOsm=295). The tissue of cauda epididymidis was minced by using sharp scissors to release spermatozoa. The spermatozoa were allowed to swim out and then incubated for 15 min in an atmosphere of 5% CO_2_ at 37^o^C, prior to determining sperm quality. For determination of sperm motility, the suspension was stirred and then one drop was placed on a pre-warmed (37^o^C) microscope slide and covered with 22×22 mm cover slip. 

The percentage of total sperm motility was estimated by visual examination at ×400 magnification using a phase contrast microscope with heated stage. For determination of sperm concentration, 10µL of the sperm suspension were transferred into an Eppendorf tube and diluted with 380 µL of D-PBS. After mixing, the sperm suspensions were counted using Neubauer counting chamber and expressed as ×10^6^/mL. Assessment of the percentage of live and dead spermatozoa was performed using a eosin-nigrosin blue staining mixture (11).

**Statistical analysis**

In the present experimental work, differences between control and treatment groups were analyzed by One-Way ANOVA while multiple comparisons were performed using Tukey’s test as post hoc. All values were given as mean±SEM and differences were considered to be significant at p<0.05. The SPSS^®^ software for windows was used for data evaluation (SPSS Inc, Chicago, IL, USA).

## Results

**Figure 1:**
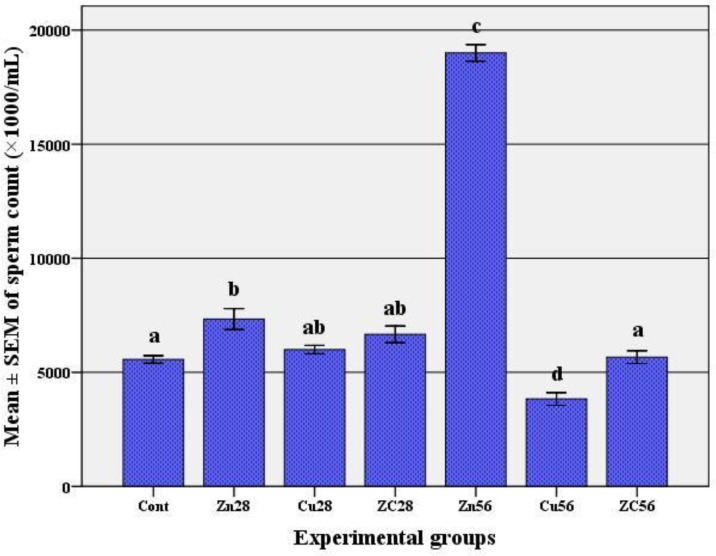
The mean±SEM of sperm count (×1000/mL) following copper consumption for 56 days and co-administration of zinc.

There was a non-significant decline in rat’s testicular weight after 28 days of copper sulfate consumption (Cu28 group, p=0.128). This testicular weight reduction, 56 days after treatment with copper (Cu56 group), showed a significant difference with the control group (p=0.002). Although a decrease was observed in the mean testicular weight of ZC group following 56 days but it was not significant in comparison to the control (Table I). Copper intake did not have any significant effect on sperm count following 28 days (Figure 1) but caused a significant decrease in the number of spermatozoa after 56 days compared to the control group (p=0.001). 

**Figure 2:**
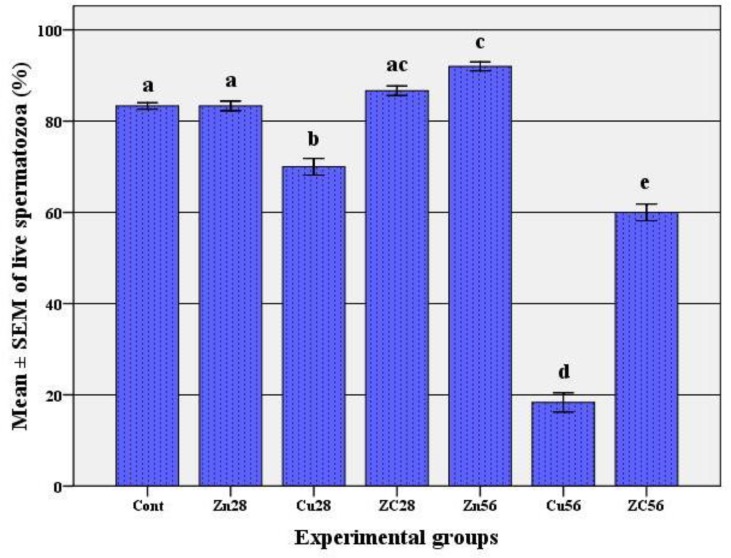
The percentage (mean±SEM) of live spermatozoa following copper consumption for 56 days and co-administration of zinc.

However, a complete recovery was observed in ZC group after 56 days. The number of spermatozoa showed a significant increase in rats that had received zinc alone in the first 4 weeks but interestingly zinc impact on sperm concentration was completely prominent after 56 days (p<0.001, Figure 1). Copper consumption by rats significantly reduced the percentage of live spermatozoa after 28 days (p<0.001, Figure 2) but its effect on decreasing the percentage of live spermatozoa was severe after 56 days (p<0.001). 

**Figure 3:**
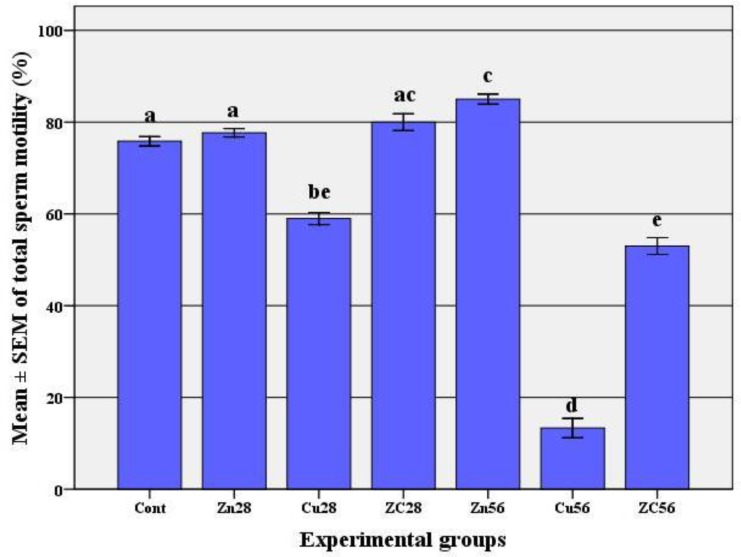
The percentage (mean±SEM) of total sperm motility following copper consumption for 56 days and co-administration of zinc.

Administration of zinc (ZC28 group) could significantly improve the adverse effect of copper on the percentage of live spermatozoa but only a partial recovery was observed after 56 days compared to Cu56 group (p<0.001) and still ZC56 group had a significant difference with the control group (p<0.001). Also, administration of zinc by itself could significantly increase the mean percentage of live spermatozoa after 56 days. The mean percentage of total sperm motility (Figure 3) decreased 28 days after copper treatment compared to the control group (p<0.001). However severe and significant impact of copper on sperm motility was observed after long term consumption (56 days). Zinc therapy completely recovered deleterious effects of copper (ZC group) in the first 28 days, so we could not see any significant difference between ZC group and the control group (p=0.328) but only a partial recovery was observed after 56 days. It means that the percentage of total sperm motility in ZC56 group was significantly higher than Cu56 group (p<0.001) and lower than the control group (p<0.001). 

Similar to the above mentioned parameters, zinc therapy after 56 days significantly improved the percentage of total sperm motility in comparison to the control group (p<0.001).

**Table I:** Comparing of the mean (± SEM) testicular weight (gr) in different groups during the study period

**Parameter**	**Control**	**Experimental groups**					
		**28 days after treatment**			**56 days after treatment**		
		**ZC**	**Cu**	**Zn**	**ZC**	**Cu**	**Zn**
No. of rats	12	6	6	6	6	6	6
Mean (±SEM)	31.40 (0.55)^a^	30.80 (0.33)^a^	29.53 (0.79)^a^	31.20 (0.38)^a^	31.16 (0.31)^a^	27.16 (0.64)^b^	28.63 (0.55)^a^

Zn: Zinc.

Cu: Copper.

ZC: Zinc and Copper

Tukey’s test as post hoc used following one-way analysis for multiple comparisons between experimental groups. ^a, b^Cu administration after 56 days showed a significant difference with the control group (p=0.002).

Cont, control; Zn28, zinc administration for 28 days; Cu28, copper consumption for 28 days; ZC28, co-administration of zinc and copper for 28 days; Zn56, zinc administration for 56 days; Cu56, copper consumption for 56 days; ZC56, co-administration of zinc and copper for 56 days. At each column different alphabet shows significant difference (p<0.05).

Cont, control; Zn28, zinc administration for 28 days; Cu28, copper consumption for 28 days; ZC28, co-administration of zinc and copper for 28 days; Zn56, zinc administration for 56 days; Cu56, copper consumption for 56 days; ZC56, co-administration of zinc and copper for 56 days. At each column different alphabet shows significant difference (p<0.05).

Cont, control; Zn28, zinc administration for 28 days; Cu28, copper consumption for 28 days; ZC28, co-administration of zinc and copper for 28 days; Zn56, zinc administration for 56 days; Cu56, copper consumption for 56 days; ZC56, co-administration of zinc and copper for 56 days. At each column different alphabet shows significant difference (p<0.05).

## Discussion

Many previous studies have reported clinical and pathological manifestations of long term Cu consumption in naturally occurring animals and similar results have been described in experimentally induced animals (1, 4, 12-16). Since the length of time from initiation of stem cell division to formation of spermatozoa is around 52 days in the rat, the chosen period of times (4 and 8 weeks) provide sufficient times to monitor the potential recovery of spermatogenesis with producing new spermatozoa in our treated groups (17). This study revealed features of Cu toxicosis in rats sperm quality specially following long term administration that is in agreement with a previous report by Sakhaee *et al* (4). 

Similar results were also reported in workers exposed to electric welding in which increased semen concentration of copper along with lowered sperm count, sperm viability and semen volume were observed (18). One of the major mechanism of cellular damage observed in chronic Cu toxicosis may be associated with Cu-induced lipid peroxidation (19). Copper is a strong oxidant, in which it could bind to cell molecules during the high load (20). Indirectly they can catalyze through Fenton/Haber-Weiss chemistry the production of cell damage as a consequence of metal-driven formation of reactive oxygen species (21, 22). 

The other possible explanation for cellular damage by Cu overload can be due to effects of Cu on cell apoptosis. Apoptosis is associated with specific morphological changes which are characterized by chromatin condensation, nuclear DNA fragmentation, cell shrinkage and membrane-enclosed cell fragment (apoptic body) formation (23). It has been shown that copper compounds delay cell-cycle progression and increase cell death in different cell cultures (24). Previous investigations provide evidence that Cu ions are capable of interacting directly with nuclear proteins and DNA causing site-specific damage (25). 

Our experiment clearly demonstrated that zinc administration has protective role against deleterious effects of Cu on sperm quality. Bremner *et al* believed that Zn could exert this protective effect in part through decreasing liver Cu content or affect Cu uptake at the intestine in supplemented animals (10). It is also possible that Zn decreases the toxicity of Cu by promoting the formation of nontoxic form of Cu. One of the proposed mechanisms of zinc’s action is its capacity to displace transition metals like Cu from binding sites which is thought to be involved in the detoxification of Cu. Zinc induces the production of metallothionein, an effective scavenger of hydroxyl radicals and it has been suggested that zinc-metallothionein complexes provide protection against free radical attack (26).

Furthermore, the sperm damage observed in our study following long term Cu administration may be associated with lipid peroxidation of sperm cell membrane or cytoplasmic organelles similar to previous report in liver (27). Therefore, another explanation for the protective effects of zinc against Cu is that zinc can compete with copper to bind to the cell membrane and decrease the production of free radicals, thus exerting a direct antioxidant action (28). 

In agreement with our study, some authors have reported that high concentration of zinc may to be associated with enhanced sperm parameters including sperm count, motility and normal morphology (7, 29-32). The sperm nucleus is composed of condensed chromatin in which the DNA is stabilized by protamines. These basic nuclear proteins, which are important for condensation and stabilization of the DNA, are held together by sulfhydryl bonds (33). Evidence has accumulated that a major fraction of Zn in the mammalian spermatozoon is located within S-S linked structures, mainly in association with sulfhydryl groups (34). Zinc stabilizes the structures of these proteins, preserves the integrity of subcellular organelles and prevents destruction of DNA by inhibiting degrading enzymes (35). On the other hand, zinc is able to affect DNA transcription and DNA and protein synthesis because serves as a cofactor for more than 80 metalloenzymes that are necessary for synthesis of DNA and proteins (36). Therefore, not only Zn has decrease adverse effects of Cu on DNA by its anti-apoptic properties but also can improve sperm quality may be through antioxidant properties (37, 38).

## Conclusion

In conclusion, our study clearly suggests that a relative protection against the poisoning effects of Cu on sperm quality following long term consumption might be obtained by Zn administration. Nevertheless, considering nontoxic and safe nature of Zn following long term administration and also its beneficial effects on sperm quality, it could be one of our choices for preventive therapies and/or improvement in semen quality after Cu toxicosis (11). 
